# Regional Burden of Anemia among Adolescent girls in India: A systematic review and meta-analysis

**DOI:** 10.3126/nje.v15i3.77656

**Published:** 2025-12-31

**Authors:** Anita Kumari, Amita Kumari, Shiv Kumar Mudgal, Vipin Patidar, Abhishek Kumar Singh, Abhimanyu Ganguly, Sanjeet Kumar Singh

**Affiliations:** 1,2,3,4,6,7 All India Institute of Medical Sciences, Deoghar, Jharkhand, India; 5 Faculty of Veterinary and Animal Sciences, RGSC, Banaras Hindu University, Varanasi, India

**Keywords:** Adolescent girls, Anemia, Burden, Indian region, Prevalence

## Abstract

**Background:**

Among Indian adolescent girl’s anemia remains a major public health concern due to rapid growth, menstrual blood loss, and nutritional deficiencies. This systematic review and meta-analysis aim to assess the prevalence and severity of anemia among Indian adolescent girls.

**Methods:**

This review (2004–2024) integrated data from 32 studies (14,053 persons) from PubMed, Embase, and Scopus, adhering to Preferred Reporting Items for Systematic reviews and Meta-Analyses (PRISMA)/ Meta-analysis Of Observational Studies in Epidemiology (MOOSE) guidelines. Observational studies with the prevalence of anemia in Indian adolescent girls based on WHO criteria were included. The data was pooled using a random-effects model, and subgroup analyses were conducted by Indian region. Heterogeneity was assessed using the I^2^ statistic.

**Results:**

The pooled anemia prevalence was 65% (95% CI: 54%–74%), showed notable regional variations. The burden was highest in East India (81%; 39%–97%), then North India (65%), West India (61%), and South India (52%). The mean hemoglobin levels varied by region, ranging from 10.24 g/dL in the East to 11.20 g/dL in the South. Mild anemia (29%) and moderate anemia (25%) were more common than severe anemia (1%). The substantial heterogeneity (I^2^=98.7%) indicated differences in socioeconomic status, diet, and healthcare access.

**Conclusion:**

Anemia affects disproportionate number of Indian adolescents’ girls, particularly in the country's east, which highlights the need for context-specific interventions. The initiatives must be linked to national programs like Anemia Mukt Bharat to ensure equitable progress towards India's public health objectives and to avoid long-term health and developmental consequences.

## Introduction

Anemia occurs when the hemoglobin level, in conjunction with the quantity and dimensions of erythrocytes, falls below a certain threshold [1]. The most common causes of anemia include malnutrition, infections, and genetic hemoglobin abnormalities [2,3]. Iron-deficiency anemia is one of the most common kinds of anemia among the other types in developing countries [4].

In India, this medical condition primarily affects children, adolescents, breastfeeding mothers, and women of reproductive age [5]. In India 253 million teenagers comprise 25.9% of the country's overall population. According to the recent Comprehensive National Nutrition Survey (CNNS), which was carried out between 2016 and 2018, the prevalence of some kind of anemia among Indian adolescents between the ages of 10 and 19 was 28%, with 12% of themdiagnosed with iron deficiency [6]. Iron deficiency anemia was found to be a major contributing factor to the decline in healthy years of life among teenagers aged 10-19 because of disability in the year 2021 [7]. The prevalence of iron deficiency and the subsequent anemia increases as adolescence begins. In adolescent girls, this is due to increased nutritional needs related to growth, which are exacerbated by the onset of menstruation in later years [2].

The amount of iron required increases two to three times, from roughly 0.7 to 0.9 mg per day during preadolescence to 1.37 to 1.88 mg per day for male adolescents and 1.40 to 3.27 mg per day for female adolescents [8]. The presence of anemia in adolescence has significant implications for many aspects of health, and the severity of anemia is directly related to almost all functional impairments associated to iron deficiency such as decreased resistance to infections, impaired cognitive and physical development, and decreased academic performance, work capacity, and physical fitness [9–11].

Research examining the prevalence of anemia among adolescent girls in India has revealed varying rates, ranging from 21% to 96% [9-10]. This significant variation may stem from differences in factors such as sample size, participant demographics, inclusion and exclusion criteria, study environments, and the methods used to measure hemoglobin levels. Additionally, the quality of individual studies varies widely. Furthermore, national statistics do not give a full picture regarding the prevalence of anemia among Indian teenage girls regional wise, overlooking significant regional variances. To address the root causes in those locations, it is essential to identify which regions have a high burden of anemia. So, this systematic review and meta-analysis were carried to figure out the regional prevalence and severity of anemia among Indian adolescent females by means of a thorough appraisal and evaluation of the quality of the academic literature. The findings could help in the development of focused interventions and regulatory plans to improve the health of Indian adolescents.

## Methodology

The research was carried out as a systematic review and meta-analysis covering the years 2004–2024. A thorough search of the literature was conducted in order to identify studies that addressed the research question ‘How prevalent is anemia among Indian adolescent girls vary across different regions?’ The search strategy included: [(Prevalence) OR (Epidemiology) OR (Frequency) AND (Anemia) OR (Iron-Deficiency anemia) OR (Macrocytic anemia) OR (Hypochromic anemia) OR (Pernicious anemia) OR (Low Hemoglobin) OR (Low Hematocrit) OR (bloodlessness) AND (Adolescent girls) OR (teenage) OR (teenaged) OR (young) AND (India)] which was employed in databases such as PubMed, Embase, and Scopus [supplementary file]. The study met the requirements of MOOSE [Meta-analysis of Observational Studies in Epidemiology) and PRISMA (Preferred Reporting Items for Systematic Reviews and Meta-Analyses] [12,13]. Additionally, under registration ID CRD42025640713, the review procedure was registered with the International Prospective Register of Systematic Reviews [PROSPERO].

### Study criteria and selection

After a preliminary review of the assigned titles and abstracts, a thorough analysis of the full-text publications was conducted. Only observational and cross-sectional studies that met the predetermined inclusion criteria and reported on the prevalence of anemia (as per WHO Criteria) were considered. studies focused on adolescent girls between the ages of 10 and 19; and population or community contexts-based studies in India were included. Researches evaluating anemia in adolescents with particular medical disorders were excluded, and the letters, abstracts, conference proceedings, reviews, and research without human participants also excluded in this meta-analysis.

Two separate reviewers examined every title that was obtained from the databases and evaluated the abstracts of relevant titles. A third author was consulted in order to settle any disagreements regarding the selection of abstracts that met the inclusion criteria. After the most recent and complete versions were verified, duplicate entries were removed. For the selected abstracts, full-text publications were acquired, and the reference lists of these articles were analyzed to find other relevant sources. The acquired full-text papers were then subjected to additional evaluation in order to determine whether or not they met the inclusion requirements.

### Qualitative evaluation of the studies and data extraction

The Joanna Briggs Institute (JBI) checklist was used to assess the studies’ quality [14]. This checklist consists of nine different questions designed to evaluate the studies' quality. Each inquiry was assigned a score of "Yes," "No," or "Unclear," culminating in an overall score that spans from 0 to 9. The scores were then divided into three categories: low quality (1–3), medium quality (4–6), and excellent quality (7–9). An extensive checklist was used to collect relevant data for data extraction, such as the author's name, the year of publication, the state of India, the study setting, the sample size, the age of the participants, the methods used to estimate hemoglobin levels, the mean hemoglobin level, the prevalence of anemia, and the severity of the anemia.

### Statistical Analysis

A thorough meta-analysis was conducted using a random-effects model with 95% confidence intervals to determine the prevalence and severity of anemia in the Indian adolescent’s population. The I^2^ statistic was utilized to evaluate heterogeneity. Subgroup analyses were conducted based on the different Indian region as well as other important factors that influence heterogeneity. The assessment of publication bias was conducted utilizing the Egger test, where a p-value below 0.05 was interpreted as indicative of potential bias. All statistical analyses were executed employing R Studio software, specifically the version 4.2.3. The functions "metamean" and "metaprop" were employed to ascertain the overall mean hemoglobin concentration and the collective prevalence of anemia among adolescents, respectively.

## Results

**Figure 1:**
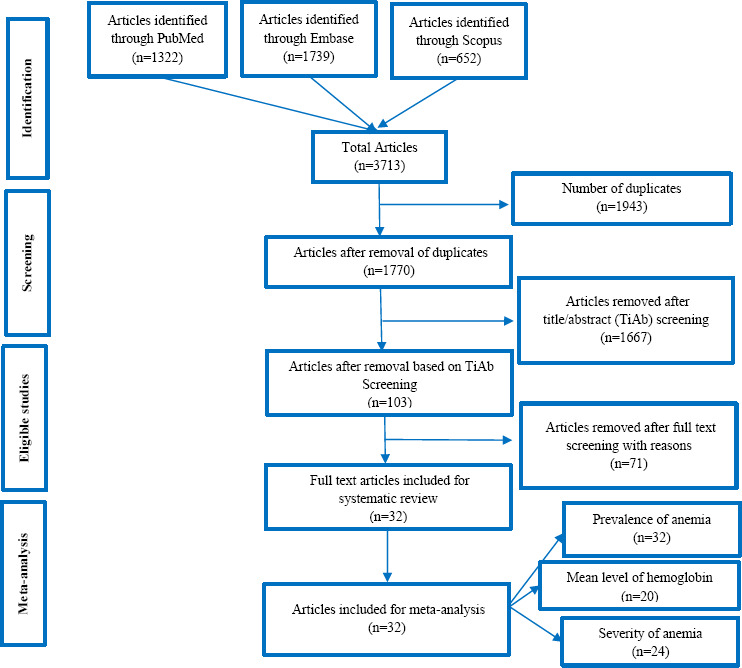
PRISMA flowchart

In total 3,713 papers were found using database searches that were part of the meta-analysis. 1943 duplicate articles were eliminated. Following an evaluation of 1770 publications' titles and abstracts, we eliminated 1667, leaving 103 for full-text analysis. 32 publications were included in the meta-analysis following a qualitative and quantitative evaluation of the 71 that did not fit the inclusion criteria. Figure 1 depicts the 2020 PRISMA flowchart.

### Characteristics of Studies

The meta-analysis included 32 studies [15-46] with 14,053 female adolescents as participants. Table 1 list out the characteristics of the studies that were part of the meta-analysis. The majority of these research were conducted in rural areas. The most common technique among the studies that described the methods used to measure hemoglobin levels was the cyanmethemoglobin technique, which was followed by Sahli's technique and HemoCue. All studies followed the WHO's hemoglobin thresholds for anemia. In terms of geographical distribution within India, the majority, 12 studies were carried out in the southern region [16,19,24,31,34,36,37,40,42,43,45, 46] with eighth, seven, and five conducted in the northern [17,18,26-29,41,44], western [15,21,23, 25,30,35,38], and eastern [20,22,32,33,39] regions, respectively. In terms of participant characteristics, the majority showed a mean age between 12.9 and 15.77 years. The prevalence of anemia was reported in all 32 studies. Additionally, 24 research [15-17,19-21,23-26,28-30,32,34-38,40-44] classified the severity of anemia into mild, moderate, and severe categories, while 20 studies [15-17,19,20,23,26,29-35,37-39,41,43,44] reported mean hemoglobin levels.

### Prevalence of Anemia among adolescent girls

**Figure 2:**
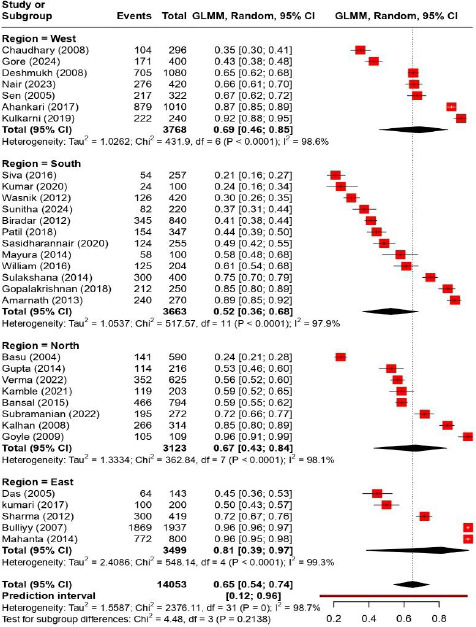
Region wise Prevalence of anemia among Indian adolescent girls.

Hemoglobin levels below 12.0 g/dL have been defined as anemia, while those between 10.0 and 11.9 g/dL were classified as mild, those between 7.0 and 9.9 g/dL as moderate, and those below 7.0 g/dL as severe. The combined estimate of the prevalence of anemia among the group of adolescent girls in India was determined to be 65% (95% CI: 54%–74%), which was calculated through all thirty-two included studies (Figure 2). The studies exhibit significant heterogeneity (I^2^=98.7%). Subgroup analysis by regional distribution shows differences, with East India showing a higher pooled prevalence of anemia among adolescent females (81% [39%, 97%]) and South India showing a considerably lower pooled prevalence of anemia (52% [36%, 68%]). The results show a large prediction interval of 0.12 to 0.96, indicating a high degree of variability.

**Figure 3:**
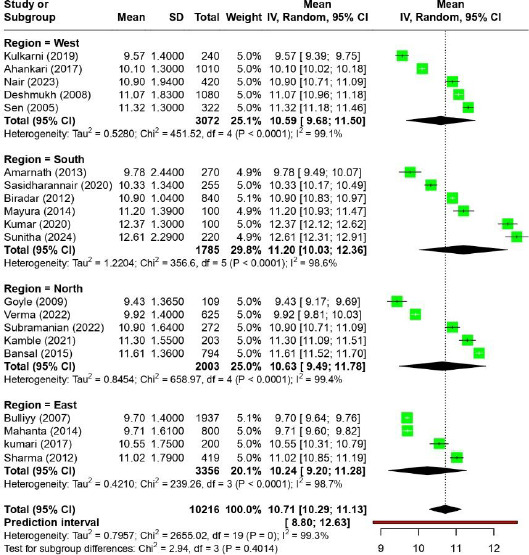
Region wise mean hemoglobin level among Indian adolescent girls.

Across twenty reviewed studies, which reported the mean hemoglobin level [15-17,19,20,23,26,29-35,37-39,41,43,44], the pooled mean hemoglobin concentration is 10.71 [10.29, 11.13] (Figure3). However, within certain geographic areas, the mean hemoglobin concentration ranges from approximately 9.43 to 12.61. The Southern regions have the highest mean values (11.20), while the Eastern regions have lowest mean values (10.24). Overall, there is considerable heterogeneity among the studies (I^2^ = 99.3%).

### Severity of anemia among adolescent girls

**Figure 4:**
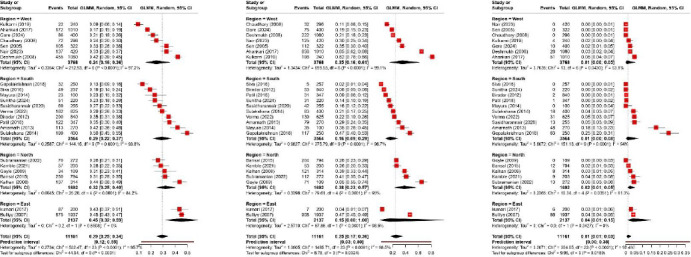
Regional wise severity of anemia (Mild, moderate and severe).

The Figure 4 presents a forest plot summarizing the severity of anemia (mild, moderate and severe) estimates across different regions (West, South, North, and East) was carried out on 24 studies [15-17,19-21,23-26,28-30,32,34-38,40-44] that reported the severity among Indian adolescent girls. The overall pooled prevalence of mild, moderate and severe anemia was 29%, 25%, and 1% respectively.

Mild anemia across all included studies is 0.29 [0.25, 0.34], with substantial heterogeneity (I^2^ = 95.7%). The subgroup analysis by region indicated that the pooled prevalence of mild anemia among adolescent girls resided in East region was highest i.e. 45% (95% CI 32%–59%; I^2^ = 0%) compared to the other region. The pooled prevalence estimates of moderate anemia was 0.25 [0.17, 0.36] with heterogeneity remains high (I^2^ = 98.5%). The subgroup analysis by region indicated that the pooled prevalence of moderate anemia among adolescent girls resided in North region was highest i.e. 38% (95% CI 23%–57%; I^2^ = 95%) compared to the other region. The overall pooled proportion of severe anemia was 0.01 [0.01, 0.03], indicating a much lower prevalence compared to mild and moderate anemia. Similarly to mild anemia the highest prevalence of severe anemia found out in east region 4% (95% CI 1%–15%; I^2^ = 0%).

### Risk of Bias

The included reports' JBI values varied from 5 to 9. Twenty-nine of the 32 studies were classified as excellent quality, and three as medium, none were classified as low quality. Complete evaluations for all included studies are provided in Supplementary File.

### Publication Bias

**Figure 5:**
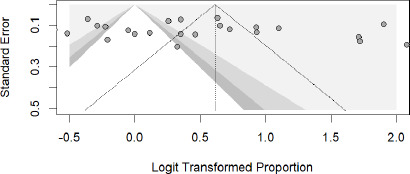
Funnel plot for publication bias.

The funnel plot was prepared to detect the possible publication bias in included studies (Figure 5). This funnel plot shows no obvious signs of publication bias as the data points are spread out fairly equally on both sides of the average, suggesting the results are balanced and unbiased. .The Egger's test also does not provide sufficient statistical support for the existence of bias (t = 1.27, p = 0.2142). The bias estimate has a considerable standard error (5.6172, SE = 4.4270), which suggests a high level of uncertainty. Additionally, a substantial degree of variation among the studies is suggested by the high heterogeneity variance (tau^2^ = 75.1695).

## Discussion

Anemia prevalence among adolescent girls in India was 65% (95% CI: 54%–74%), based on 32 studies, underscoring the need for public health interventions. This aligns with a meta-analysis by Daniel [47], which reported a 65.7% prevalence, but contrasts with Akbari [48], who found a lower prevalence of 13.9% (95% CI: 10.8%–17.1%) among Iranian children and adolescents, and 7.9% (95% CI: 4.1%–11.7%) and 8.5% (95% CI: 6.1%–10.8%) among Iranian men and women, respectively. The disparity may be because of differences in race and ethnicity. Factors contributing to India's higher anemia rates might be menstrual bleeding, rapid growth during adolescence, socio-economic challenges, limited healthcare access, and rural living conditions with restricted food options [48-49].

The eastern area has a highest prevalence of anemia among Indian adolescent girls (81%). The finding goes contrary to earlier findings that indicated anemia affects around half of the people in the eastern region [50]. This high prevalence might be due to widespread poverty, lack of access to quality food and lack of quality health services. On the other hand, the prevalence rate was lower in southern India (52%), which might be due to higher dietary intake, urbanization or use of better dietary measures [51]. The significant regional differences associated with these health problems reflect the interaction between social, nutritional and cultural factors. These regional differences support the concept that anemia is not only a medical condition but also a marker of inequality.

The prevalence of mild (29%) and moderate (25%) cases of anemia is also concerning even though the prevalence of severe anemia is relatively low (1%). Educational outcomes, physical strength, and intellectual functioning are important in adolescent development, and even mild anemia can affect these outcomes [52]. The severity of the disease was higher in the eastern region (45% mild, 4% severe), reflecting a lack of timely diagnosis and inadequate treatment. These results contradict the commonly held belief that only severe anemia should be treated. Early iron deficiency still requires treatment to prevent significant growth in children and adolescents [10]. Anemia can be exacerbated by factors such as less intake of iron rich diet, parasitic infections, menstrual blood loss, and lack of knowledge about proper nutrition. Certain dietary factors, such as low bioavailability of iron in vegetarians and gender differences in food preferences also increase the risk of anemia [9,10].

The impact and effectiveness of existing Indian programs such as POSHAN Abhiyaan and Anemia Mukt Bharat need to be carefully evaluated and region-specific strategies need to be implemented [53,54]. The strategy may include promoting iron-rich foods, integrating deworming programs in schools, improving nutrition education to detect malnutrition and anemia, and improving nutritional supplementation in schools and colleges. To address this issue and ensure that young women not only survive but thrive, a multi-sectoral strategy that integrates social support, education and health is needed. As India achieves key demographic and economic goals, the health of young people must be prioritized in development plans.

To our best knowledge this is one of the first meta-analysis to systematically quantify regional disparities in anemia prevalence among Indian adolescent girls, this study's main strengths are its extensive scope, which is reinforced by strict adherence to PRISMA/MOOSE guidelines. In-depth region wise subgroup analyses, large sample size, and the results have important policy implications since they pinpoint high-burden region also key strength of this review. However, the study has several limitations including limited generalizability in urban setting as most of study conducted among rural population, differences in hemoglobin measurement methods (cyanomethemoglobin and HemoCue), lack of detailed information on other dietary and nutritional factors, and substantial high heterogenicity included study.

## Conclusion

The prevalence of anemia in Indian teenage girls is surprisingly high with notable regional variations, making it a significant public health concern. Lower rates in South India raise the possibility of learning from dietary and programmatic practices, but the East Indian burden points to structural differences in nutrition, healthcare access, and socioeconomic development. The national efforts like Anemia Mukt Bharat Abhiyaan must be combined with locally specific intervention to tackle this disease.

## Figures and Tables

**Table 1:** Summary table (Burden of anemia among adolescent girls in India: A systematic review and meta-analysis).

Author	State	Setting	Age (mean ± SD) or age group	Sample size (N)	Method of Hemoglobin estimation	Mean Hemoglobin	Prevalence of anemia n (%)	Mild n (%)	Moderate n (%)	Severe n (%)	Quality
**Ahankari (2017) [15]**	Maharashtra	Rural	13-17 Year	1010	Sahli’s hemometer method.	10.1±1.3 g/dL	879 (87%)	172(17)	656(65)	51(5)	Excellent
**Amarnath (2013) [16]**	Andhra Pradesh	Rural	10–19 year	270	Sahli's method	9.78±2.44 g/dl	240 (88.9)	113 (41.9)	79 (29.3)	48 (17.8)	Excellent
**Bansal (2015) [17]**	Delhi	Urban	11–18 year	794	Cyanmethemoglobin method.	11.61±1.36 g/dl	466 (58.7)	250 (31.5)	204 (25.7)	12 (1.5)	Excellent
**Basu (2004) [18]**	Chandigarh	Rural and Urban	12-18 year	590	Cyanmethemoglobin method	NA	141 (23.9)	NA	NA	NA	Excellent
**Biradar (2012) [19]**	Karnataka	Rural	10-19 Year	840	Automated cell counter (Beckman Coulter AC T diff 2).	10.9±1.04 g/dL	345 (41.1)	290 (34.5)	53 (6.3)	2 (0.2)	Excellent
**Bulliyy (2007) [20]**	Orissa	NA	15.77±2.20	1937	Cyanmethemoglobin method	9.7±1.4 g/dL	1869 (96.5)	875 (45.2)	908 (46.9)	86 (4.4)	Excellent
**Chaudhary (2008) [21]**	Maharashtra	Urban	10-19 Year	296	Cyanmethemoglobin method	NA	104 (35.1)	72 (24.3)	32 (10.8)	0 (0)	Excellent
**Das (2005) [22]**	West Bengal	Rural	10-19 years	143	NA	NA	64 (44.8)	NA	NA	NA	Medium
**Deshmukh (2008) [23]**	Maharashtra	Rural and Urban	10-19 years	1080	Standard Procedure	11.07±1.83 g/dl	705 (65.3)	455 (42.1)	222 (20.6)	28 (2.6)	Excellent
**Gopalakrishnan (2018) [24]**	Tamil Nadu	Urban	13-18 years	250	Portable digital hemoglobin meter	NA	212 (84.8)	32 (12.8)	117 (46.8)	63 (25.2)	Excellent
**Gore (2024) [25]**	Western region	Rural	14 years	400	HemoCue 201	NA	171 (42.8)	86 (21.5)	75 (18.8)	10 (2.5)	Excellent
**Goyle (2009) [26]**	Rajasthan	Urban	10-16 years	109	Cyanmethemoglobin method using Hemocor-D kit	9.43±1.365 g/dl	105 (96.3)	34 (31.2)	71 (65.1)	0 (0)	Medium
**Gupta (2014) [27]**	Uttar Pradesh	Urban	10-19 years	216	Direct Cyanmethemoglobin method	NA	114 (52.8)	NA	NA	NA	Excellent
**Kalhan (2008) [28]**	Haryana	NA	10-19 years	314	NA	NA	266 (84.7)	137 (43.6)	121 (38.5)	8 (2.5)	Excellent
**Kamble (2021) [29]**	Delhi	Urban	14.6±1.18	203	HemoCue 201	11.3±1.55 g/dl	119 (58.6)	57 (28.1)	53 (26.1)	9 (4.4)	Excellent
**Kulkarni (2019) [30]**	Maharashtra	Rural	14.5±1.62	240	Cyanmethemoglobin method.	9.57±1.4 g/dl	222 (92.5)	22 (9.2)	196 (81.7)	4 (1.7)	Excellent
**Kumar (2020) [31]**	Karnataka	Rural	14-16 years	100	Automated 6-part differential cell counter.	12.37±1.3 g/dl	24(24)	NA	NA	NA	Excellent
**kumari (2017) [32]**	Bihar	Urban	10-19 years	200	Sahli’s method	10.55±1.75 g/dl	100(50)	87 (43.5)	7 (3.5)	6(3)	Excellent
**Mahanta (2014) [33]**	Assam	Rural	14.8 ± 2.3	800	Cyan-meth-hemoglobin method	9.71±1.61 g/dl	772 (96.5)	NA	NA	NA	Excellent
**Mayura (2014) [34]**	Puducherry	Rural	15.63± 2.0	100	Test strips (Hemoglobin Colour Scale 4)	11.2±1.39 g/dl	58(58)	23(23)	35(35)	0 (0)	Excellent
**Nair (2023) [35]**	Maharashtra	Rural	14.01±2.57	420	Cell Counter named Mythic Haematoanalyzer.	10.90±1.94 g/dl	276 (65.7)	137 (32.6)	125 (29.8)	0 (0)	Excellent
**Patil (2018) [36]**	Karnataka	Urban	14.34±1.8	347	Hemoglobin cuvette.	NA	154 (44.4)	122 (35.2)	31 (8.9)	1 (0.3)	Excellent
**Sasidharannair (2020) [37]**	Tamil Nadu	Rural	10–19 year	255	Five-part automated cell counter (Beckman Coulter AC T diff 2).	10.33±1.34 g/dl	124 (48.6)	69 (27.1)	42 (16.5)	13 (5.1)	Excellent
**Sen (2005) [38]**	Gujarat	Urban	09–14 year	322	Cyanmethemoglobin method	11.32±1.3 g/dL	217 (67.4)	105 (32.6)	112 (34.8)	0 (0)	Excellent
**Sharma (2012) [39]**	Assam	Rural	13.62 ±1.1	419	Cyanmethemoglobin method	11.02±1.79 g/dl	300 (71.6)	NA	NA	NA	Excellent
**Siva (2016) [40]**	Kerala	Rural	13.39 ± 2.3	257	Auto-analyzer SYSMAX 800i.	NA	54(21)	49 (19.1)	5 (1.9)	0 (0)	Excellent
**Subramanian (2022) [41]**	Haryana	Rural	15.5±1.8	272	Digital hemoglobinometer method (Hemocue201+)	10.9±1.64 g/dl	195 (71.7)	70 (25.7)	112 (41.2)	13 (4.8)	Excellent
**Sulakshana (2014) [42]**	Karnataka	Rural	12.9±2.06	400	Cyanmethemoglobin method	NA	300(75)	199 (49.8)	83 (20.8)	18 (4.5)	Excellent
**Sunitha (2024) [43]**	Tamil Nadu	Rural	13.8±1.4	220	Whatmann filter paper for haemoglobin estimation.	12.61±2.29 g/dl	82 (37.3)	51 (23.2)	31 (14.1)	0 (0)	Excellent
**Verma (2022) [44]**	Rajasthan	Rural	15.54±2.72	625	HemoCue (Hb 201).	9.92±1.40 g/dl	352 (56.3)	182 (29.1)	139 (22.2)	31(5)	Excellent
**Wasnik (2012) [45]**	Andhra Pradesh	Urban	10-15 years	420	NA	NA	126(30)	NA	NA	NA	Medium
**William (2016) [46]**	Tamil Nadu	NA	15-18 years	204	Calorimetric method.	NA	125 (61.3)	NA	NA	NA	Excellent

SD=Standard deviation; n=number of participants; NA= Not available;
